# Determination of Adulteration Content in Extra Virgin Olive Oil Using FT-NIR Spectroscopy Combined with the BOSS–PLS Algorithm

**DOI:** 10.3390/molecules24112134

**Published:** 2019-06-06

**Authors:** Hui Jiang, Quansheng Chen

**Affiliations:** 1 School of Electrical and Information Engineering, Jiangsu University, Zhenjiang 212013, China; 2 School of Food and Biological Engineering, Jiangsu University, Zhenjiang 212013, China

**Keywords:** bootstrapping soft shrinkage, partial least squares, extra virgin olive oil, adulteration, FT-NIR spectroscopy

## Abstract

This work applied the FT-NIR spectroscopy technique with the aid of chemometrics algorithms to determine the adulteration content of extra virgin olive oil (EVOO). Informative spectral wavenumbers were obtained by the use of a novel variable selection algorithm of bootstrapping soft shrinkage (BOSS) during partial least-squares (PLS) modeling. Then, a PLS model was finally constructed using the best variable subset obtained by the BOSS algorithm to quantitative determine doping concentrations in EVOO. The results showed that the optimal variable subset including 15 wavenumbers was selected by the BOSS algorithm in the full-spectrum region according to the first local lowest value of the root-mean-square error of cross validation (RMSECV), which was 1.4487 % v/v. Compared with the optimal models of full-spectrum PLS, competitive adaptive reweighted sampling PLS (CARS–PLS), Monte Carlo uninformative variable elimination PLS (MCUVE–PLS), and iteratively retaining informative variables PLS (IRIV–PLS), the BOSS–PLS model achieved better results, with the coefficient of determination (R^2^) of prediction being 0.9922, and the root-mean-square error of prediction (RMSEP) being 1.4889 % v/v in the prediction process. The results obtained indicated that the FT-NIR spectroscopy technique has the potential to perform a rapid quantitative analysis of the adulteration content of EVOO, and the BOSS algorithm showed its superiority in informative wavenumbers selection.

## 1. Introduction

With the rising prices of cooking oil, greedy traders and suppliers may resort to unethical practices, such as mixing low-value cooking oil with high-value cooking oil [1]. The consumers cannot detect these low-value, inexpensive ingredients in cooking oils, so they pay more for them. Extra virgin olive oil (EVOO) is native to the Mediterranean area, is known as “the gold of liquids”, “the queen of plant oils”, and “the Mediterranean nectar”, and is an established Chinese consumer favorite [2]. The consumption of the EVOO has increased in recent years. However, the production of EVOO is not enough to cope with the growing consumer demand in China because of the demanding production conditions of EVOO. Therefore, EVOO adulteration has spread in the Chinese market. Adulteration not only causes confusion in the edible oil market but also violates the rights of consumers. Therefore, a fast and effective analytical method of EVOO adulteration is required to assist government’s regulations.

Fourier transform near-infrared (FT-NIR) molecular spectroscopy is a technique widely applied in food quality analysis [3,4,5,6] that can provide abundant information about the chemical composition and molecular structure of various food substances. In addition, this technology also has the advantages of being non-destructive, fast, low-cost, with good reproducibility and broad application prospects. Recently, the FT-NIR spectroscopy technique has been extensively used in quality and safety analysis of EVOO [7,8,9]. In addition, other molecular spectroscopy techniques, such as fluorescence spectroscopy [10,11,12], infrared spectroscopy [13,14,15], Raman spectroscopy [16,17,18], and nuclear magnetic resonance spectroscopy [19,20], have good applications in the analysis of EVOO adulteration. With the technological developments, the amount of spectral data acquired is increasingly large because of the improvement of instrument resolution. Therefore, the selection of spectral characteristic wavenumbers plays an important role in spectral model development. Moreover, more and more researchers have proved that the selection of characteristic wavenumbers in the multivariable model calibration can not only improve the prediction performance of the chemometrics model but also enhance the interpretability of the model [21,22,23,24].

Partial least-square (PLS) regression is a statistical method related to principal component regression (PCR), which is to search a linear regression model by projecting predicted variables and observed variables into a new state space [25]. Because of the advantages of variable selection, many PLS-based feature variable selection algorithms have been developed [26], for example, the variable importance in projection (VIP) score [27], the successive projections algorithm (SPA) [28], the uninformative variable elimination (UVE) algorithm [29], and the selectivity ratio (SR) [30]. These methods were developed on the basis of the criteria of variable weights or regression coefficients. Additionally, some other feature wavenumber selection methods based on model population analysis (MPA) strategies have been developed [31], for instance, the iteratively retaining informative variables (IRIV) [32], the variable iterative space shrinkage approach (VISSA) [33,34], the variable combination population analysis (VCPA) [35], and the bootstrapping soft shrinkage (BOSS) [36]. Compared with IRIV, VISSA, and VCPA, an important feature of the BOSS algorithm is the introduction of weighted bootstrap sampling (WBS) criteria that the other three algorithms do not consider. Furthermore, different from other bootstrap-based algorithms, the BOSS algorithm performs the bootstrap criteria in the variable space, while other algorithms perform the criteria in the sample space. Thus, in this study, the BOSS algorithm was applied for the wavenumber selection of spectral data of EVOO doped samples.

The aim of this study was to verify the feasibility of establishing an improved and reliable reduced spectral model which can directly and quantitatively determine the doping content of EVOOs by their spectra. The feature wavenumbers were first selected by the BOSS algorithm, and a detection model based on the PLS regression using the selected wavenumbers by the BOSS algorithm was built. Finally, the performance of the reduced BOSS–PLS model was compared with the performances of the other three commonly used reduced models (i.e., competitive adaptive reweighted sampling PLS (CARS–PLS), Monte Carlo uninformative variable elimination PLS (MCUVE–PLS), and iteratively retaining informative variables PLS (IRIV–PLS)).

## 2. Results

### 2.1. Variable Selection by the BOSS Algorithm

In this study, the informative wavenumbers were firstly selected by using the BOSS algorithm during PLS modeling. A five-fold cross validation was used for the optimization of relevant parameters, and the optimal variables were finally determined according to the first local lowest root-mean-square error of cross validation (RMSECV) value. Before running the BOSS algorithm, the number of bootstrap sampling was set to 1000, and the maximum number of principal components (PCs) was set to 15. In this study, in order to verify the repeatability and stability of the algorithm, the approach was conducted repeatedly 10 times, and the best results were recorded.

**Figure 1:**
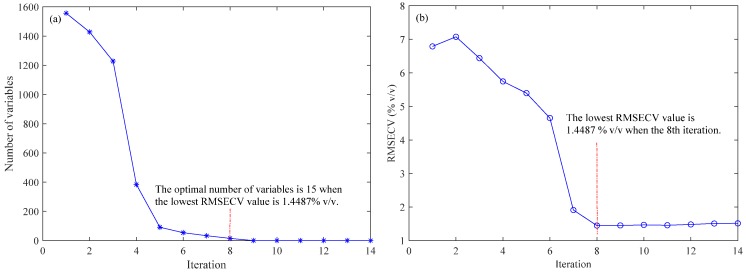
Evolution of the number of variables (**a**) and root-mean-square error of cross validation (RMSECV) (**b**) in each iteration of the sub-models using the bootstrapping soft shrinkage (BOSS) algorithm.

Figure 1 shows the evolution of the variables and the value of RMSECV in each iteration of sub-models during the run of the BOSS algorithm. The number of wavenumbers selected decreased smoothly with iteration of the BOSS algorithm. The initial number of wavenumbers obtained was 1557 from the full spectrum. As can be seen in Figure 1a, the number of variables selected gradually decreased and became 1 after 14 iterations. Meanwhile, as can be seen in Figure 1b, the values of RMSECV in the sub-models decreased with the increase of the iteration number, reached the minimum value at the eighth iteration, and then started to rise slowly. The best variable subset was finally achieved in the eighth iteration, and the optimal number of wavenumbers selected was 15 at the eighth iteration, according to the first local lowest RMSECV, which was 1.4487 % v/v.

**Figure 2:**
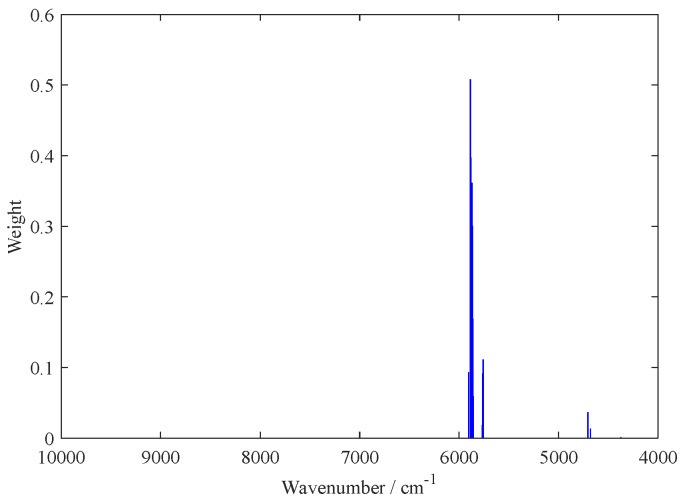
The weights of the variables in the optimal sub-model at the eighth iteration using the BOSS algorithm.

Figure 2 shows the weights and the wavenumbers distribution in the full spectrum of the 15 variables selected at the eighth iteration of the sub-models; it shows the 15 variables selected with their respective weights and the variable with the largest weight and highest importance. By investigating the results in Figure 2, the most informative wavenumbers were finally obtained at around 5900 cm^−1^. Thus, the 15 variables selected by the BOSS algorithm constituted the best variable subsets for building the final PLS model.

### 2.2. Results of the PLS Model

**Figure 3:**
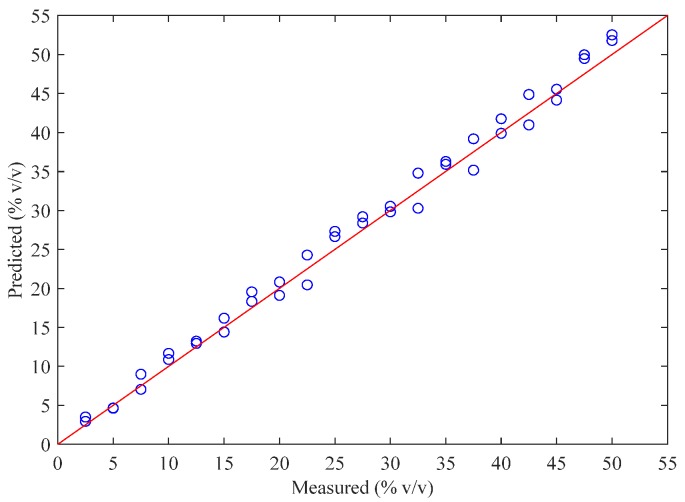
Reference-measured versus FT-NIR-predicted doping concentration of extra virgin olive oil (EVOO) in the validation set.

The optimal PLS model was built using the 15 wavenumbers selected by the BOSS algorithm when three PLS factors were included. The value of RMSECV was 1.4487 % v/v, and the R^2^ was 0.9908 in the calibration set. The predictive accuracy and generalization performance of the constructed model were evaluated using the independent samples from the validation set. The result of the root-mean-square error of prediction (RMSEP) was 1.4889 % v/v, and the R^2^ was 0.9922 in the validation set which, as shown in Figure 3.

## 3. Discussion

In order to show the advantages of the BOSS algorithm in terms of wavenumber selection, it was compared with other three high-performance approaches for wavenumber selection, i.e., CARS, MCUVE, and IRIV. The best results of PLS models based on variables selected from different variable selection algorithms are shown in Table 1. The results in Table 1 show that the prediction accuracy of the PLS model could be improved by the four wavenumber selection algorithms with respect to the full-spectrum PLS model. Moreover, compared with the CARS–PLS model, the MCUVE–PLS model, and the IRIV–PLS model, the BOSS–PLS model achieved better results not only in the calibration process but also in the validation process. The main reason is that, quite likely, the BOSS algorithm combines the strategies of soft shrinkage, MPA, and WBS and makes full use of the regression coefficient information.

Also, the BOSS algorithm adopts the soft shrinkage strategy to select informative variables. Compared with the method of variable selection based on the hard shrinkage strategy, such as CARS and MCUVE, which delete less informative wavenumbers directly, the soft shrink strategy allocates smaller weights to wavenumbers with less information. However, these wavenumbers can still participate in the sub-models’ construction for further evaluation considerations in the next iteration. Thus, the advantage of the soft shrink strategy is that it is able to reduce the risk of removing characteristic variables during the iteration and to choose the optimal variable subsets with better prediction ability.

The best variable subset is finally obtained by the BOSS algorithm on the basis of the criteria of the MPA combined with those of the WBS. Concretely, the sub-models are obtained in terms of the weight of each variable by the BOSS algorithm. The weight of each wavenumber is determined according to the value of the regression coefficients of multiple PLS sub-models by using the MPA strategy, rather than by using a single full-spectrum model. Then, the WBS strategy is used to stepwise update the weight of the wavenumbers selected so that the variable space can be compressed better. Thus, the BOSS algorithm considers all possible combinations of the selected wavenumbers, which is reasonable because the best number of variable subsets obtained is unknown before and during wavenumbers selection.

## 4. Materials and Methods

### 4.1. Sample Preparation and Division

In this study, extra virgin olive oil, peanut oil, sunflower seed oil, soybean oil, sesame oil, and maize oil were purchased in local supermarkets. In the experiments, peanut oil, sunflower seed oil, soybean oil, sesame oil, and corn oil were used as adulterating oils, which would be added separately to the EVOO to prepare the samples to be tested. That is to say, the adulterated oil samples were prepared including only two kinds of edible oil, namely, the EVOO and one of adulterating oils. The specific preparation process is reported below.

The doped oil samples were prepared using the EVOO and one of the adulterating oils. The volume fraction of each adulterated oil ranged from 2.5 to 50% v/v, increasing by 2.5% v/v volume fraction. Thus, 100 samples could be obtained in the experiment process.

In this study, the 100 samples were divided into two subsets. One was the calibration set, which was adopted to construct the prediction model, the other was the validation set, which was applied to verify the accuracy and generalization performance of the model. In order to meet the statistical requirements, three samples at the same doping concentration were randomly selected and put into the calibration set during sample division. Thus, there were 60 samples in the calibration set and 40 samples in the validation set. Because the adulterated samples obtained in this study only contained two kinds of edible oils, the calibration model established in this study can only be used to quantitatively detect one adulterated oil mixed with EVOO.

### 4.2. FT-NIR Spectra Acquisition

In this study, the NIR spectra of the doped samples were collected in transmission mode by means of an Antaris II NIR spectrophotometer (Thermo Scientific Co., Waltham, MA, USA). The number of spectral scanning was set to 32, and the spectral resolution was set to 4 cm^−1^. The range of spectral scanning was set from 10,000 cm^−1^ to 4000 cm^−1^. Thus, the original spectrum of each doped sample contained 1557 wavenumbers (i.e., 1557 wavelength variables). The absorbance data were stored as Log (1/T), T being the transmittance.

In spectral collection, each doped sample was first placed in a cuvette with a diameter of 6.0 mm, and then in the sampling chamber of the spectrometer for original spectral collection. The spectra of each doped sample were collected three times, and the mean values of the three measured spectra were taken as the original NIR spectra of the sample. When the spectra were collected, the laboratory temperature maintained at 25 °C.

### 4.3. Spectra Preprocessing

**Figure 4:**
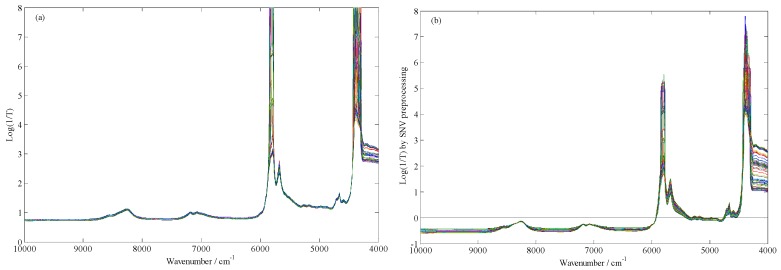
The original FT-NIR spectra (**a**) and the standard normal variate (SNV) preprocessing FT-NIR spectra (**b**) of all adulterated EVOO samples.

Figure 4a shows the raw FT-NIR spectra of all collected samples. As can be seen from Figure 4a, the spectra obtained contained not only useful sample information but also certain noise information, even overflow occurred in some wavenumbers. In order to eliminate the influence of these adverse factors, it was necessary to adopt appropriate methods to preprocess the spectra obtained before multivariable model calibration. Standard normal variate (SNV) transformation, which can be used to eliminate not only the baseline drift of diffuse reflectance spectrum but also the overflow phenomenon of diffuse reflectance spectrum, is mainly used to eliminate the influence of surface scattering and optical path change on diffuse reflectance spectra. Therefore, in this study, the SNV method was adopted to pretreat the spectra obtained, and the FT-NIR spectra after SNV preprocessing are presented in Figure 4b.

### 4.4. Data Analyses Methods

The BOSS algorithm applied here, which can be used to select the characteristic variables in the presence of collinearity, was described by Deng et al. [36]. The BOSS algorithm is based on a favorable criterion of shrinkage and utilizes the information of regression coefficients instead of the traditional hard shrinkage strategy. The BOSS algorithm, which is based on the bootstrap sampling (BSS) [37] and WBS [38] techniques, was used to determine the random combination wavenumbers and to establish the sub-models. The MPA was applied to extract informative variable subsets from the sub-models developed on the basis of PLS regression. The specific process of the BOSS algorithm was as follows:

In the process of spectral data analysis, suppose the spectral data matrix is *X*, of size *N* × *P*, which includes *N* samples and *P* wavenumbers, and a vector *Y*, of size *N* × 1, which represents the reference measurements.

Step 1, *K* subsets were generated in a variable space by the BSS. In each subset, one of many redundant variables remained by the BSS to extract characteristic variables. In the step, all wavenumbers were treated equally so that they had the same probability of being selected into the variable subset. That is to say, each variable had the same weights (*w*)

Step 2, the *K* sub-model of PLS were first developed by the data from the subsets selected. Then, the cross-validation RMSECV of each sub-model was calculated, each sub-model was sorted from smallest to largest, according to the RMSECV value, and the sub-model ranked in the top 10% was extracted.

Step 3, the regression coefficients of each sub-model extracted was calculated. By normalizing each regression vector, all elements in the regression vector were transformed into the absolute value of unit length. The new weights of the variable selected were then obtained according to the following summation formula:

(1) $$w_{i}=\sum_{i=1}^{K}b_{i,k}$$

where *K* represents the number of sub-models that are extracted, and $b_{i,k}$ is the absolute value of the normalized regression coefficients for the *i*th wavenumber in the *k*th sub-model.

Step 4, the WBS was used to generate some new subsets based on the new weight of each variable selected, and the number of substitution wavenumbers in the WBS was obtained according to the average number of wavenumbers selected in the last step.

Step 5, steps 2 to 4 were repeatedly conducted until the number of wavenumbers selected in the renewed variable subset equaled one, and the variable subset was finally selected according to the lowest value of the RMSECV during the iterations as the best variable subset.

### 4.5. Model Evaluation

The prediction and generalization performances of the models were examined by a five-fold cross validation and an independent validation set. The values of the RMSECV, RMSEP, and coefficient of determination (R^2^) were used as measures for model performance evaluation. RMSECV, RMSEP, and R^2^ are given by the expressions

(2) $$\mathrm{RMSECV}=\sqrt{\frac{\sum_{i=1}^{n}{\left(\hat{y_{\backslash i}}-y_{i}\right)}^{2}}{n}}$$

(3) $$\mathrm{RMSEP}=\sqrt{\frac{\sum_{i=1}^{n}{\left(y_{i}-\hat{y_{i}}\right)}^{2}}{n}}$$

(4) $$R^{2}=1-\frac{\sum_{i=1}^{n}{\left(y_{i}-\hat{y_{i}}\right)}^{2}}{\sum_{i=1}^{n}{\left(y_{i}-\bar{y_{i}}\right)}^{2}}$$

For RMSECV, *n* is the number of samples in the calibration set, *y_i_* is the reference measurement value from the *i*th sample, and $\hat{y_{\backslash i}}$ is the estimated value of the *i*th sample, when the model is constructed with the removed *i*th sample. For RMSEP, *n* is the number of samples in validation set, *y_i_* is the reference measurement value of the *i*th sample in the validation set, and $\hat{y_{i}}$ is the estimated value of the *i*th sample in the validation set. For R^2^, *n* is the number of samples, *y_i_* is the reference measurement value from the *i*th sample, $\hat{y_{i}}$ is the estimated value of the *i*th sample, and $\bar{y_{i}}$ is the mean of all samples.

### 4.6. Software

All algorithms were implemented in Matlab R2018a (Mathworks, Natick, MA, USA) under Windows 10. The Matlab codes for implementing BOSS are freely available on the website: http://www.mathworks.com/matlabcentral/fileexchange/52770-boss.

## 5. Conclusions

The results obtained in this study show the potentials of FT-NIR spectroscopy in the detection of adulterations in EVOO. The BOSS algorithm combines the strategies of soft shrinkage, MPA, and WBS and could be used to extract the informative wavenumbers from the full-spectrum. The BOSS–PLS model revealed its superiority with respect to the full-spectrum PLS, CARS–PLS, MCUVE–PLS, and IVIR–PLS models. It can be concluded that the FT-NIR spectroscopy technique is an effective tool for the determination of EVOO adulteration and has a good guiding significance for the evaluation of EVOO quality. Moreover, the BOSS algorithm is a promising wavenumbers selection algorithm in chemometrics analysis, which can improve the prediction performance of calibration models.

## Figures and Tables

**Table 1:** Results of different partial least-square (PLS) models for the prediction of doping concentrations in EVOO. CARS: competitive adaptive reweighted sampling; MCUVE: Monte Carlo uninformative variable elimination; IRIV: iteratively retaining informative variables.

Models	Selected Wavenumbers (cm^−1^)	Number of Variables	PLS Factors	Calibration Set		Validation Set	
				R^2^	RMSECV	R^2^	RMSEP
PLS	9999.10-3999.64	1557	6	0.9421	3.4618	0.9599	3.2520
CARS-PLS	4192.49; 4242.63; 4261.92; 4578.18; 4593.61; 4655.32; 4659.18; 4666.89; 4670.75; 4674.60; 4682.32; 4690.03; 5746.83; 5754.55; 5758.40; 5766.12; 5858.68; 5862.54; 5870.25; 5874.11; 5877.97; 5881.82; 5885.68; 5889.54; 5897.25; 5901.11; 5912.68; 5920.39; 5935.82; 8234.55	30	4	0.9617	2.9647	0.9683	2.7664
MCUVE-PLS	4373.76; 4412.33; 4566.61; 4593.61 4612.89; 4632.18; 4647.61 4670.75; 4690.03; 4709.32; 5750.69; 5762.26; 5777.69; 5866.40; 5885.68; 5904.97; 5924.25; 5939.68; 6001.39; 6028.39; 8238.41; 8253.84; 8261.55; 8265.41	24	3	0.9694	2.6828	0.9778	2.3232
IRIV-PLS	4373.76; 4412.33; 5750.69; 5754.55; 5758.40; 5762.26; 5769.97; 5773.83; 5777.69; 5854.83; 5858.68; 5862.54; 5866.40; 5874.11	14	2	0.9901	1.4877	0.9887	1.8471
BOSS-PLS	4373.76; 4678.46; 4705.46; 5758.40; 5762.26; 5766.12; 5777.69; 5858.68; 5862.54; 5866.40; 5870.25; 5877.97; 5881.82; 5885.68; 5904.97	15	3	0.9908	1.4487	0.9922	1.4889
